# Prize Essay—Medical Association of the State of Alabama

**Published:** 1875-06

**Authors:** 


Prize Essay—Medical Association of the State of Alabama.—At the Annual Session of this Association, held at Montgomery during the second week in April, Dr. S. D. Seelye, after some remarks regarding the increasing frequency with which cases of Bright's Disease are brought under medical care in this section, the unsatisfactory though extensive literature of the subject, and the generally fatal prognosis which we are obliged to give, rendering it one of the opprobria of the profession; and believing that there is still some hidden light that may be made to illumine the subject if attention and inquiry are turned especially to it; proposed to place in the hands of this Association one hundred dollars for the best treatise on this disease, under the following regulations, viz:

1. Competion for the prize to be open to the whole country.
2. A committee of five to be appointed to adjudicate upon the essays presented.
3. All essays to be forwarded to the chairman of said committee, on or before the first day of February, 1876, and to be accompanied by a sealed letter containing name and address of the author, which letter shall not be opened until after the adjudication is made.

4. The Prize Essay to be the property of this Association, and to be published in the Annual Volume of Transactions, and all unsuccessful papers to be returned to the address of the authors; honorable mention being made of any deemed of especial merit.

5. If none of the essays presented are deemed worthy of the prize, the committee shall have the privilege of rejecting all, in which case the competion shall be open for another year.

6. The prize shall be one hundred dollars in currency, with a certificate of this Association suitably inscribed and bearing the seal of the Association; or it will be wrought into a Gold Medal or Plate, with a suitable legend and a fac simile of the seal of the Association engraved thereon, to be of the full value of one hundred dollars, less the price of manufacture, at the option of the successful author.

7. The adjudication shall be publicly announced at the next annual meeting of Association, to be held at Mobile during the second week in April, 1776.

The above proposition having been accepted by this Association, it now invites from the thinkers and investigators of the profession at large, the generous competition which its author invokes.

Committee of Adjudication.—Dr. Jerome Cochrane, Mobile, Ala., Chairman,; Dr. J. B. Gaston, Montgomery, Ala.; Dr. W. H. Anderson, Mobile, Ala.; Dr. Peter Bryce, Tuskaloosa, Ala.

Benj. H. Riggs, M.D.,
Sec'y M. A. S. A., Selma, Ala.

---

Caution—Ziemssen's Cyclopædia of the Practice of Medicine. As this great work progresses, it is possible—from some subscribers breaking up their sets, or from other causes—that occasional odd volumes may be offered for sale. Those who desire the complete work are warned against purchasing these, as the publishers do not engage to supply parts of sets. Every subscription must be for the entire work. No volumes will be sold separately.

Wm. Wood & Co., Publishers,
27 Great Jones Street, New York.

